# Renewing Our Focus on Vulnerable Populations Among People Living with HIV

**DOI:** 10.3390/tropicalmed9110278

**Published:** 2024-11-14

**Authors:** James Ayieko, Marguerite Thorp, Musie Ghebremichael

**Affiliations:** 1 Centre for Microbiology Research, Kenya Medical Research Institute, Nairobi 54840-00200, Kenya; 2 Division of Infectious Diseases, David Geffen School of Medicine, University of California Los Angeles, Los Angeles, CA 90095, USA; 3 Harvard Medical School and Ragon Institute of Massachusetts Institute of Technology, Massachusetts General Hospital and Harvard, Cambridge, MA 02139, USA; musie_ghebremichael@dfci.harvard.edu

## 1. Introduction

The global HIV landscape has changed over the past few decades, with great milestones achieved in both HIV treatment and prevention. Access to lifesaving antiretroviral therapy (ART) has markedly expanded, with a total of 30.7 million (27 million–31.9 million) out of 39.9 million (36.1 million–44.6 million) people living with HIV accessing the medication in 2023 [1]. Continued expansion of access to, initiation of, and adherence to treatment is crucial in achieving control of the HIV pandemic, given the strong evidence that treatment is prevention [2]. Despite these marked advances, 28% of people living with HIV (PLHIV) are reported to be virally unsuppressed [1]. Viral non-suppression is associated with increased risk of progression to AIDS and portends poor outcomes for PLHIV [3,4]. Additionally, viral non-suppression increases the risk of onward transmission of HIV, reversing the gains made in combating the pandemic [3]. The risk of viral non-suppression is greater in certain groups. This Special Issue focuses on exploring HIV support, care, and treatment for vulnerable populations, or those at elevated risk of viral non-suppression and poor health outcomes.

We solicited articles on this topic and received submissions from diverse settings and authors of different backgrounds and training. The interest and importance of this topic are revealed in the diversity of articles that were submitted and the disciplines that showed interest. This Special Issue contains ten articles that advance our understanding of vulnerable populations, challenge the current thinking about vulnerable populations, and propose bold interventions to address the barriers to HIV care engagement throughout the cascade.

The articles in this Special Issue bring to the fore three critical questions about vulnerable groups: What makes one vulnerable? What are the threats to care engagement for vulnerable people? And what health care system changes are needed to accommodate vulnerable people? These questions must be addressed to improve outcomes among vulnerable groups, especially to design interventions that address their concerns.

## 2. What Makes One Vulnerable?

Our definition of vulnerable populations for this Special Issue is ‘populations that are at a higher risk of poor outcomes or have circumstances that compromise meaningful HIVcare engagement’. In this context, ‘meaningful HIV care engagement’ means involvement in regular HIV care, receiving and adhering to effective antiretroviral therapy—which is necessary to fully benefit from antiretroviral medications. Vulnerable populations can include children, adolescents, and young adults; elderly individuals; and stigmatized groups such as people who inject drugs, sex workers, men who have sex with men, and transgender individuals. Our definition assumes that the circumstances defining vulnerability are dynamic, though they may be modifiable or non-modifiable. In cases where circumstances are modifiable, reducing vulnerability would require modification of the circumstances.

Whereas proposed definitions of vulnerable populations consider categories of PLHIV in ‘buckets’, in this Special Issue, Brotherton M, a legal practitioner, argues for the expansion of the definition beyond specific groups. She argues that all PLHIV should be considered a vulnerable group deserving of prioritized access to health care and services [5]. Her opinion article anchors its argument on human rights principles and the inherent threat of exclusion if all PLHIV are not considered vulnerable. The article acknowledges the public health position that there should be particular sensitivity for intersectional issues such as poverty, discrimination, and disability among those living with HIV. However, considering all PLHIV as vulnerable enables one to appreciate the challenges faced by PLHIV even in the absence of other vulnerabilities.

It is important to note that mobility and migration, a critical function of livelihood, influence disease subtypes and strains within a population. Sarker et al. report genetic diversity and transmission dynamics of non-C subtypes of HIV seen in Bangladeshi returned migrant workers [6]. The identification of multiple subtypes indicates that some variants in Bangladesh may have accompanied migrant workers returning from different geographical areas. This analysis describes disease transmission patterns and attempts to identify sexual behavior patterns responsible for disease spread. This is important for understanding patterns of disease distribution, the development of prevention interventions, and the implementation of care programs for mobile populations living with HIV. Mobility impacts HIV engagement profoundly, and health systems must understand and adapt to human mobility and its attendant complexities [7].

## 3. What Are the Threats to Care Engagement for Vulnerable People?

We must understand the factors that threaten care engagement to identify solutions. At four points in the care cascade, PLHIV encounter major barriers to successful ART treatment: delay in or failure to initiate ART, lack of persistence with therapy, poor adherence to ART, and viral resistance to antiretroviral medication [8]. These barriers are exacerbated by circumstances that define vulnerable populations. For instance, a child who is dependent on a caregiver for their engagement in HIV care could experience the entire spectrum of these barriers, from delay in ART initiation to viral resistance resulting in poor outcomes. Arbune M et al. describe a case study that highlights this reality and missed opportunities in improving outcomes among an especially vulnerable person living with HIV [9].

Failures in our care delivery systems define an important threat to successful treatment outcomes. A “one-size-fits-all” approach failed when a multi-month drug dispensing intervention was tried among children living with HIV in Mozambique [10]. In this work described by Meque et al., MMD was provided to PLHIV, including children due to COVID-19 restrictions aimed at reducing personal contact, but the outcomes among children were not favorable. This provides an important counterbalance to the preponderance of benefits of MMD among adults; pediatric care may not be improved by rapid transition to 6-MMD.

## 4. What Health Care System Changes Are Needed to Accommodate Vulnerable People?

Health care delivery systems must be deliberate to improve health outcomes among vulnerable populations. As previously highlighted, a one-size-fits-all approach is likely to be unsuccessful. Patient-centered care is a promising solution to challenges around care engagement with tailored interventions to improve outcomes along the entire care cascade. Patient-centered interventions aimed at adolescents, youth and mobile populations have led to improvement in viral suppression as well as mental health in these vulnerable groups [11,12,13].

Social support is crucial to address threats that may be unique or more pronounced to specific groups, such as stigma among youth going through life transitions/stages. In a pre-post study analysis, Thurman et al. describe the effects of an adolescent support group that demonstrated a significant impact on knowledge of HIV, adherence to medications, contraception use, and downstream effects such as reduced school absences [14]. Though the authors label their effect sizes as small, a 10-percentage point increase in ALYHIV achieving 95% adherence would have tremendous impact on both health outcomes and the epidemic.

Holistic patient care in the treatment of PLHIV, seen as a tenet of patient-centered care, is required for optimal service delivery, especially among vulnerable populations. Msefula et al. show a depression prevalence of 23% among AYPLHIV in an urban facility in Malawi [15]. Rates of depression were higher among ALYHIV who were responsible for their own income, who consumed alcohol, and who reported one or more sexual partners in bivariate analysis. They further observe that lack of basic needs was a chief explanation for depressive symptoms, as well as stigma and lack of access to psychosocial screening and care. This study reveals the multi-layered factors that hinder care engagement and how these factors collectively result in poor outcomes. Focus on ART alone would be futile if the health care system does not address other issues such as mental health and social support systems.

## 5. Conclusions

In conclusion, vulnerable populations merit greater focus if the fight against AIDS is to be won. Interventions to serve vulnerable populations will yield desired results only if they are designed to be responsive to the subjects. A holistic, patient-centered approach to addressing issues facing vulnerable populations is necessary to optimize treatment outcomes among these groups.
